# *Clostridium perfringens* α-toxin impairs granulocyte colony-stimulating factor receptor-mediated granulocyte production while triggering septic shock

**DOI:** 10.1038/s42003-019-0280-2

**Published:** 2019-01-31

**Authors:** Masaya Takehara, Soshi Seike, Yuuta Sonobe, Hiroto Bandou, Saki Yokoyama, Teruhisa Takagishi, Kazuaki Miyamoto, Keiko Kobayashi, Masahiro Nagahama

**Affiliations:** Department of Microbiology, Faculty of Pharmaceutical Sciences, Tokushima Bunri University, Yamashiro-cho, Tokushima, 770-8514 Japan

## Abstract

During bacterial infection, granulocyte colony-stimulating factor (G-CSF) is produced and accelerates neutrophil production from their progenitors. This process, termed granulopoiesis, strengthens host defense, but *Clostridium perfringens* α-toxin impairs granulopoiesis via an unknown mechanism. Here, we tested whether G-CSF accounts for the α-toxin-mediated impairment of granulopoiesis. We find that α-toxin dramatically accelerates G-CSF production from endothelial cells in response to Toll-like receptor 2 (TLR2) agonists through activation of the c-Jun N-terminal kinase (JNK) signaling pathway. Meanwhile, α-toxin inhibits G-CSF-mediated cell proliferation of Ly-6G^+^ neutrophils by inducing degradation of G-CSF receptor (G-CSFR). During sepsis, administration of α-toxin promotes lethality and tissue injury accompanied by accelerated production of inflammatory cytokines in a TLR4-dependent manner. Together, our results illustrate that α-toxin disturbs G-CSF-mediated granulopoiesis by reducing the expression of G-CSFR on neutrophils while augmenting septic shock due to excess inflammatory cytokine release, which provides a new mechanism to explain how pathogenic bacteria modulate the host immune system.

## Abstract (web summary)

Takehara et al. show that *Clostridium perfringens* alpha-toxin impairs granulocyte colony-stimulating factor receptor-mediated granulocyte production while enhancing host inflammation. This study identifies dual mechanisms, whereby bacterial toxin simultaneously cripples and overstimulates host immunity to its demise.

## Introduction

Neutrophils play an important role in the innate immune system by eliminating pathogenic bacteria^1–3^. During steady-state conditions, a certain number of neutrophils are maintained, whereas granulopoiesis is accelerated during bacterial infection to strengthen host defense^4–6^. Granulocyte colony-stimulating factor (G-CSF), which is a glycoprotein, has been reported to mediate these so-called steady-state and emergency granulopoiesis responses.

G-CSF influences neutrophil differentiation and proliferation. Steady-state and infection-driven granulopoiesis are impaired in G-CSF-deficient mice^7,8^. In addition, G-CSF receptor (G-CSFR)-deficient mice represent a similar phenotype^9^. During Gram-negative bacterial infection, endothelial cells play a key role in sensing lipopolysaccharide (LPS) from the infecting bacteria through a Toll-like receptor 4 (TLR4)- and myeloid differentiation factor 88 (MyD88)-dependent pathway, leading to the increased release of G-CSF into the systemic circulation^10^. The secreted G-CSF acts on myeloid precursors and accelerates the proliferation and differentiation of neutrophils in bone marrow and spleen^10–12^. Additionally, TLR2 is a pivotal receptor for the recognition of Gram-positive bacteria^13^. Recently, we reported that peptidoglycan (PGN), which is a TLR2 ligand^14,15^, promotes the secretion of G-CSF from monocytes and endothelial cells, leading to the acceleration of granulopoiesis^16^. The finding suggested that bacterial recognition by TLR2 facilitates granulopoiesis during Gram-positive bacterial infection. Thus granulopoiesis is precisely regulated to defeat pathogenic bacteria, which contributes to the preservation of the host innate immune system. Nevertheless, some bacteria can still cause life-threatening infections through serious neutropenia, and the mechanism behind this is less well understood.

*Clostridium perfringens* type A is a Gram-positive, anaerobic bacterium that causes life-threatening gas gangrene in humans^17,18^. *C. perfringens*-induced gas gangrene is accompanied by the destruction of muscle, shock, multiple organ failure, and death in affected patients^19^. *C. perfringens* infection progresses rapidly, and death precedes diagnosis in some patients. Furthermore, it has been reported that polymorphonuclear leukocytes are absent in *C. perfringens*-infected tissue^20,21^. These findings suggested that *C. perfringens* can evade host innate immunity by influencing neutrophils. α-Toxin (phospholipase C), which is a major virulence factor during *C. perfringens* type A infection^22^, mediates the formation of platelet-leukocyte aggregates^23,24^, and the aggregates impede neutrophil extravasation^25^. In addition, perfringolysin O, a cholesterol-dependent cytolysin^26,27^, has direct cytotoxic effects on polymorphonuclear leukocytes and macrophages^28–30^. The findings demonstrated that the toxins produced by *C. perfringens* type A interfere with neutrophil functions. Moreover, we recently reported that α-toxin inhibits neutrophil differentiation to impair the innate immune system^31^. α-Toxin has two enzyme activities, phospholipase C (PLC) and sphingomyelinase (SMase)^22^, and these activities are involved in the α-toxin-mediated blockage of neutrophil differentiation^31^. *Staphylococcus aureus* SMase disrupts cholesterol-rich plasma membrane microdomains, lipid rafts, in human lymphocytes^32^. Similarly, α-toxin disturbs lipid raft integrity in neutrophils, which is related to the blockage of neutrophil differentiation^33^. However, the detailed molecular mechanism remains unclear.

Previously, we reported that α-toxin upregulates the release of a chemotactic cytokine, interleukin-8 (IL-8), through activation of the endogenous PLC and TrkA signaling pathway from A549 human lung adenocarcinoma cells^34,35^. In addition, α-toxin reduces the production of tumor necrosis factor-α (TNF-α) from LPS-stimulated RAW 264.7 murine macrophages^36^. These results suggested that α-toxin affects host inflammatory responses by modulating the expression of cytokines. In this study, to elucidate the mechanism of α-toxin-induced inhibition of granulopoiesis, we tested whether α-toxin obstructs the production of G-CSF and/or G-CSFR-mediated cell growth. Here we demonstrate that α-toxin disturbed G-CSF-mediated granulopoiesis by reducing the expression of G-CSFR on neutrophils and augmented the inflammatory response due to excess inflammatory cytokine release during LPS-induced sepsis, which provides a new mechanism to explain how pathogenic bacteria modulate the host immune system.

## Results

### α-Toxin augments G-CSF endothelial expression via JNK activation

**Fig. 1:**
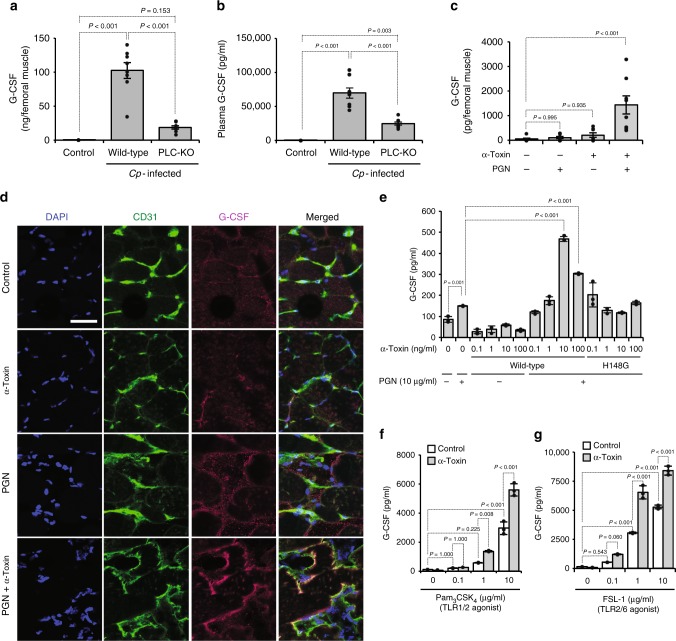
*C. perfringens* α-toxin accelerates the production of granulocyte colony-stimulating factor (G-CSF) in endothelial cells. **a**, **b** Mice were intramuscularly injected with 1 × 10^7^ colony-forming units (CFUs) of *C. perfringens* Strain 13 (wild-type), phospholipase C knockout (PLC-KO), or TGY medium as a control (Control). At 24 h after infection, G-CSF levels in the infected muscle (**a**, *n* = 8 per condition) or plasma (**b**, *n* = 8 per condition) were measured by enzyme-linked immunosorbent assay. **c**, **d** Mice were injected intramuscularly with 20 ng of purified α-toxin and 100 μg of peptidoglycan (PGN). At 24 h after the administration, G-CSF levels in the muscle were determined (**c**, *n* = 8 per condition), or the muscle was subjected to immunohistochemical analysis with antibodies against CD31 and G-CSF (**d**). Scale bar, 40 µm. **e** Human umbilical vein endothelial cells (HUVECs) were cultured for 24 h in the presence or absence of the indicated concentrations of α-toxin (wild-type) or a variant α-toxin (H148G) and 10 μg ml^−1^ PGN. G-CSF levels in the culture medium were determined (*n* = 3 per condition). **f**, **g** HUVECs were cultured for 24 h in the presence or absence of 10 ng ml^−1^ α-toxin and the indicated concentration of Toll-like receptor 2 (TLR2) agonist, Pam_3_CSK_4_ (**f**, *n* = 3 per condition) or fibroblast-stimulating lipopeptide (FSL-1) (**g**, *n* = 3 per condition), and G-CSF levels in the culture medium were determined. One-way analysis of variance was employed to assess statistical significance. Values are mean ± standard error (**a**–**c**) or standard deviation (**e**–**g**)

Generally, the production of G-CSF is accelerated during bacterial infection, but it has not been elucidated whether *C. perfringens* infection affects G-CSF production in a mouse model. The G-CSF levels in mice intramuscularly injected with *C. perfringens* type A (wild type (WT)) were greatly elevated in the infected tissue and peripheral blood, whereas the elevations were attenuated in a *plc* gene-knockout mutant (PLC-KO) *C. perfringens*-infected mice (Fig. 1a, b). Previously, we reported that PLC-KO-infected mice were more efficient at reducing the load of *C. perfringens* compared with Strain 13 (WT)-infected mice^31^, but positive relationship between G-CSF production and bacterial colony-forming units (CFUs) in Strain 13-infected muscles was not observed (Supplementary Figure 1). Next, we tested whether α-toxin was sufficient to increase the production of G-CSF. An intramuscular injection of purified α-toxin alone had no effect on the peripheral levels of G-CSF, but simultaneous administration of PGN and α-toxin notably increased G-CSF levels in a dose-dependent manner (Fig. 1c, Supplementary Figure 2). Endothelial cells play main roles in the sensing of bacterial components and the production of G-CSF during bacterial infection^10^. Immunohistochemical analysis revealed that CD31^+^ endothelial cells produced G-CSF in mice simultaneously treated with PGN and α-toxin (Fig. 1d). Correspondingly, treatment with α-toxin alone had no promoting effect on the production of G-CSF in human umbilical vein endothelial cells (HUVECs), whereas the toxin increased G-CSF production in the presence of PGN (Fig. 1e). The higher concentration of α-toxin (100 ng ml^−1^) represented a weaker effect on the elevation of G-CSF production compared with the lower concentration (10 ng ml^−1^), which was caused by a cytotoxic effect of α-toxin on HUVECs (Supplementary Figure 3). A variant α-toxin (H148G) lacking PLC and SMase activities lost the ability to amplify G-CSF production, demonstrating that α-toxin affected endothelial cells in its enzymatic activity-dependent manner (Fig. 1e). Notably, the amplification of G-CSF production by α-toxin was not seen in isolated bone marrow-derived Ly-6G^−^Ly-6C^+^ monocytes, meaning that the effect of α-toxin is cell-type dependent (Supplementary Figure 4). TLR2 forms heterodimers with either TLR1 or TLR6 and recognizes a wide range of bacterial components, including lipopeptides and lipoteichoic acids^37,38^. α-Toxin accelerated G-CSF production in HUVECs treated with a synthetic peptide agonist against TLR2/TLR1, Pam_3_CSK_4_^39^, or TLR2/TLR6, fibroblast-stimulating lipopeptide^40^, respectively (Fig. 1f, g). Thus α-toxin augmented the production of G-CSF from endothelial cells only in the presence of TLR2 agonists, suggesting that α-toxin enhances TLR2 signaling.

**Fig. 2:**
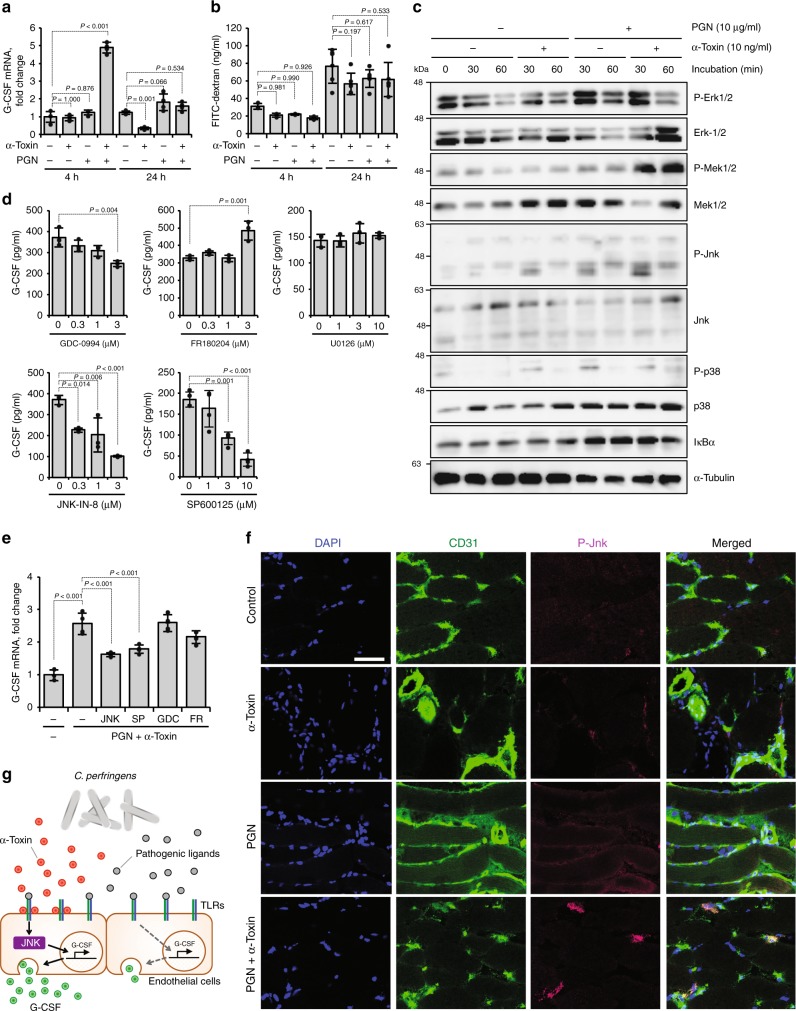
α-Toxin augments the production of granulocyte colony-stimulating factor (G-CSF) through activation of the JNK signaling pathway. **a** Human umbilical vein endothelial cells (HUVECs) were cultured for 4 or 24 h in the presence or absence of 10 ng ml^−1^ α-toxin and 10 μg ml^−1^ peptidoglycan (PGN). Total RNA was extracted and subjected to real-time reverse transcriptase PCR (RT-PCR) using a specific primer set for G-CSF (*n* = 4 per condition). **b** HUVECs were cultured for 4 (*n* = 3 per condition) or 24 h (*n* = 6 per condition) in the presence or absence of 10 ng ml^−1^ α-toxin and 10 μg ml^−1^ PGN. The concentrations of exocytosed fluorescein isothiocyanate (FITC)-dextran in the culture medium were quantified. **c** HUVECs were cultured for 30 or 60 min in the presence or absence of α-toxin and PGN, and whole-cell extracts were analyzed by immunoblotting with specific antibodies. Representative blots are shown of three independent experiments, and raw gel images are available in Supplementary Figure 9. **d** HUVECs were cultured for 24 h in the presence of 10 ng ml^−1^ α-toxin and 10 μg ml^−1^ PGN and in the presence or absence of the indicated concentration of GDC-0994 (*n* = 3 per condition), FR180204 (*n* = 3 per condition), U0126 (*n* = 3 per condition), JNK-IN-8 (*n* = 3 per condition), or SP600125 (*n* = 4 per condition). G-CSF levels in the culture medium were determined. **e** HUVECs were cultured for 24 h in the presence or absence of 10 ng ml^−1^ α-toxin, 10 μg ml^−1^ PGN, and 3 μM GDC-0994 (GDC), 3 μM FR180204 (FR), 3 μM JNK-IN-8 (JNK), or 10 μM SP600125 (SP). Total RNA was extracted and subjected to real-time RT-PCR using a specific primer set for G-CSF (*n* = 3–4 per condition). **f** Mice were injected intramuscularly with 20 ng of purified α-toxin and 100 μg of PGN, and the muscle was subjected to immunohistochemical analysis with antibodies against CD31 and phospho-JNK (P-JNK). Scale bar, 50 µm. **g** Model of accelerated production of G-CSF in a *C. perfringens*-infected host. One-way analysis of variance was employed to assess statistical significance. Values are mean ± standard deviation. Similar results were obtained in two independent experiments

To elucidate the mechanism how α-toxin promotes G-CSF production, we measured G-CSF mRNA expression levels in HUVECs treated with α-toxin and PGN and found that simultaneous treatment with both agents temporarily upregulated G-CSF expression in a dose-dependent manner (Fig. 2a, Supplementary Figure 5). In addition, α-toxin had no effect on the efflux of fluorescence-labeled dextran from HUVECs, demonstrating that α-toxin does not accelerate exocytosis. These results suggested that α-toxin promotes de novo synthesis of G-CSF. Stimulation of TLR2 activates mitogen-activated protein kinases (MAPKs) and nuclear factor kappa B (NF-κB)^41^. Simultaneous treatment of HUVECs with α-toxin and PGN upregulated phosphorylation levels of extracellular signal-regulated kinase 1/2 (ERK1/2), mitogen-activated protein kinase kinase 1/2 (MEK1/2) and c-Jun N-terminal kinase (JNK), but α-toxin plus PGN did not induce degradation of IκBα (Fig. 2c). Inhibitors against JNK (JNK-IN-8 and SP600125) but not those against ERK1/2 and MEK1/2 (GDC-0994, FR180204, and U0126) profoundly reduced G-CSF production from HUVECs treated with α-toxin and PGN with no apparent cytotoxicity (Fig. 2d, Supplementary Figure 6). In additon, inhibitors against JNK suppressed the upregulated mRNA expression levels of G-CSF (Fig. 2e). To test whether α-toxin activates the JNK signaling pathway in mice intramuscularly injected with α-toxin and PGN, immunohistochemical analysis was performed. Single administration of α-toxin or PGN did not induce phosphorylation of JNK, respectively, whereas simultaneous administration of these reagents increased the phosphorylation in CD31^+^ endothelial cells (Fig. 2f). Together, our results illustrated that α-toxin upregulates pathogenic ligand-induced TLR signaling, such as the JNK signaling pathway, during *C. perfringens* infection leading to increased production of G-CSF from endothelial cells (Fig. 2g).

**Fig. 3:**
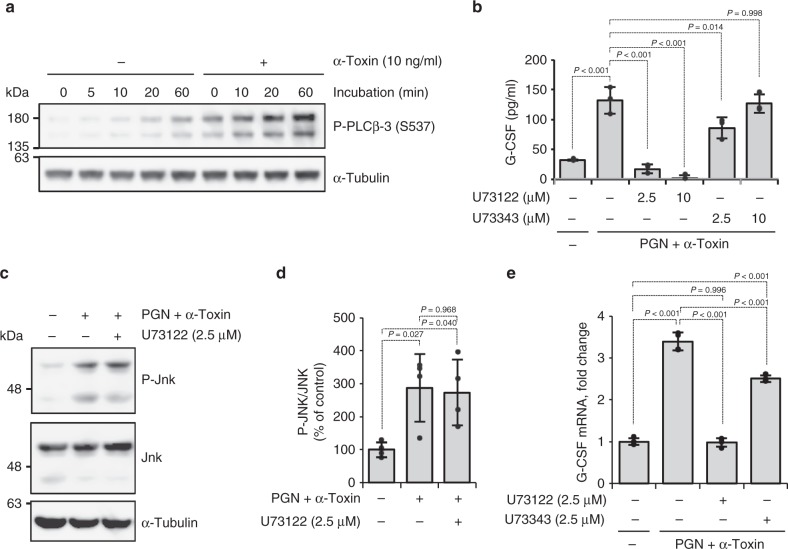
Activation of endogenous phospholipase C (PLC) by α-toxin contributes to increased production of granulocyte colony-stimulating factor (G-CSF). **a** Human umbilical vein endothelial cells (HUVECs) were cultured in the presence or absence of α-toxin, and whole-cell extracts were analyzed at the indicated time by immunoblotting with specific antibodies. Representative blots are shown of three independent experiments, raw gel images are available in Supplementary Figure 9. **b**–**e** HUVECs were cultured for 24 h (**b**), 1 h (**c**, **d**), or 4 h (**e**) in the presence of 10 ng ml^−1^ α-toxin and 10 μg ml^−1^ peptidoglycan (PGN) and in the presence or absence of the indicated concentration of U73122 or U73343. G-CSF levels in the culture medium were determined (**b**, *n* = 3 per condition). Whole-cell extracts were analyzed by immunoblotting with specific antibodies and the density of bands was measured (**c**, **d**, *n* = 4 per condition). Total RNA was extracted and subjected to real-time reverse transcriptase PCR using a specific primer set for G-CSF (**e**, *n* = 4 per condition). Representative blots are shown of four independent experiments, raw gel images are available in Supplementary Figure 9 (**c**). One-way analysis of variance was employed to assess statistical significance. Values are mean ± standard deviation. Similar results were obtained in two independent experiments

Previously, we reported that α-toxin stimulates the formation of 15-*N*-nervonoyl sphingosine (C_24:1_-ceramide) by activating endogenous SMase in sheep erythrocytes^42^. To test whether ceramide affects the expression of G-CSF, we treated HUVECs with PGN and cell-permeable ceramide analog (C_2_-ceramide) and found that C_2_-ceramide has no impact on the production of G-CSF (Supplementary Figure 7). Next, we tested whether α-toxin activates endogenous phosphoinositide-specific PLC (PI-PLC). In a previous report, α-toxin was reported to activate endogenous PLCγ-1 to promote IL-8 release in A549 cells^34^. We investigated the activation of endogenous PLCs by α-toxin. α-Toxin did not affect phosphorylation of PLCγ-1 and PLCγ-2, but it greatly increased phosphorylation of PLCβ-3 (Fig. 3a, Supplementary Figure 8). A PI-PLC inhibitor (U73122), but not its inactive analog (U73343), decreased G-CSF secretion upregulated by treatment with α-toxin and PGN (Fig. 3b). U73122 did not affect the increase of phosphorylation of JNK by α-toxin and PGN, whereas it completely inhibited the upregulation of G-CSF mRNA expression by α-toxin and PGN (Fig. 3c–e). These results indicate that the activation of endogenous PLCβ-3 by α-toxin contributes to increased production of G-CSF from endothelial cells, but it is independent of JNK signaling pathway.

### α-Toxin desensitizes neutrophils to G-CSF by reducing its receptor levels

**Fig. 4:**
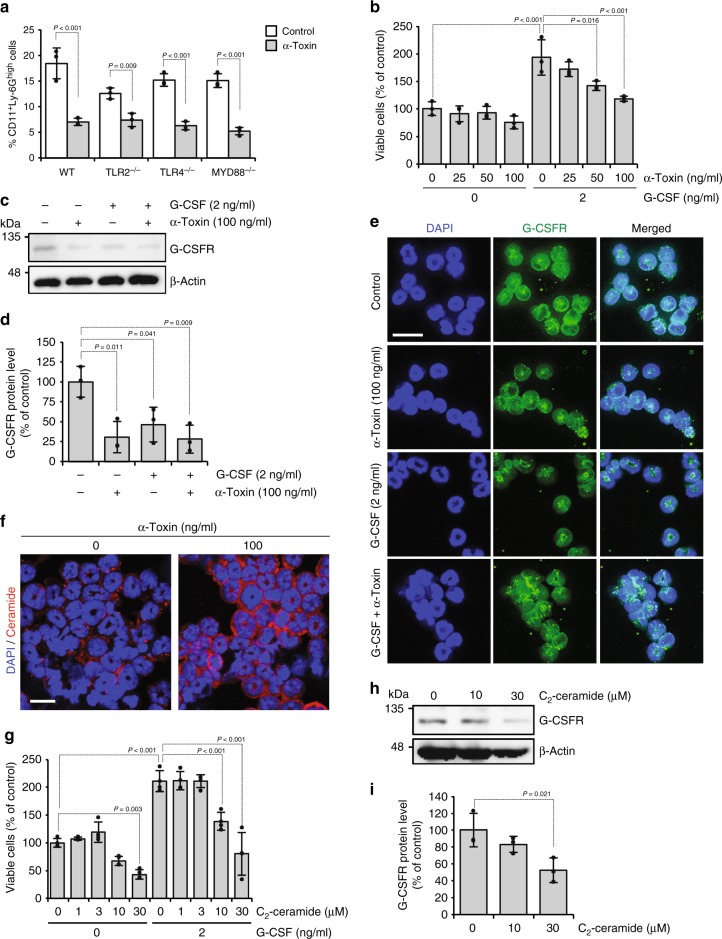
α-Toxin makes neutrophils insensitive to granulocyte colony-stimulating factor (G-CSF). **a** Bone marrow cells derived from wild-type (WT), *Tlr2*^−*/*−^ (TLR2^−/−^), *Tlr4*^−*/*−^ (TLR4^−/−^), and *Myd88*^−*/*−^ (MYD88^−/−^) mice were cultured for 24 h in the presence or absence (Control) of 100 ng ml^−1^ α-toxin (α-Toxin), and flow cytometric analysis was performed. The frequency of CD11b^+^Ly-6G^high^ neutrophils is shown (*n* = 3 per condition). **b**–**e** Magnetically isolated Ly-6G^+^ cells were cultured for 24 h in the presence or absence of the indicated concentrations of G-CSF and α-toxin. The viable cells were determined using a Cell Counting Kit-8 (**b**, *n* = 3 per condition). Whole-cell extracts were analyzed by immunoblotting with specific antibodies against G-CSFR and β-actin, and the density of bands was measured (**c**, **d**, *n* = 3 per condition). Representative blots are shown of three independent experiments, raw gel images are available in Supplementary Figure 9 (**c**). Localization of G-CSFR was monitored by an immunostaining assay (**e**). Scale bar, 20 µm. **f** Ly-6G^+^ cells were cultured for 30 min in the presence or absence of 100 ng ml^−1^ α-toxin. The cells were subjected to immunofluorescence analysis of ceramide. Scale bar, 20 µm. **g**–**i** Ly-6G^+^ cells were cultured for 24 h in the presence or absence of the indicated concentrations of G-CSF and C_2_-ceramide. The viable cells were determined using a Cell Counting Kit-8 (**g**, *n* = 4 per condition). Whole-cell extracts were analyzed by immunoblotting with specific antibodies against G-CSFR and β-actin, and the density of bands was measured (**h**, **i**, *n* = 3 per condition). Representative blots are shown of three independent experiments, raw gel images are available in Supplementary Figure 9 (**h**). One-way analysis of variance was employed to assess statistical significance. Values are mean ± standard deviation. Similar results were obtained in two independent experiments

Because α-toxin has no inhibitory effect on the production of G-CSF in vivo and in vitro, we supposed that α-toxin directly affected neutrophils and blocked their differentiation. Our previous results showing that α-toxin decreased the number of CD11b^+^ Ly-6G/6C^high^ differentiated neutrophils in isolated Ly-6G/6C^+^ neutrophils in vitro supported this notion^31^. Additionally, we reported that disturbance of cell membrane lipid rafts by α-toxin is related to the blockage of neutrophil differentiation^33^. Then we focused on plasma membrane receptors and related signaling. From this study, α-toxin promoted pathogenic ligand-induced TLR signaling. Figure 4a shows that α-toxin decreased the population of CD11b^+^Ly-6G^high^ differentiated neutrophils in bone marrow cells (BMCs) derived from WT, *Tlr2*^−*/*−^ (TLR2^−/−^), *Tlr4*^−*/*−^ (TLR4^−/−^), and *Myd88*^−*/*−^ (MYD88^−/−^) mice, indicating that TLR signaling is not involved in differentiation blockage by the toxin (Fig. 4a). Next, we tested whether α-toxin affects G-CSFR signaling. Cell proliferation of isolated Ly-6G^+^ neutrophils from BMCs was promoted by treatment with recombinant mouse G-CSF, and α-toxin dose-dependently inhibited the accelerated cell proliferation (Fig. 4b). Previously, we reported that α-toxin inhibits differentiation of mouse neutrophils at 100 ng ml^−1^ (ref. ^31^), which is consistent with the result showing that maximal inhibition of the G-CSF-mediated cell proliferation requires concentration of 100 ng ml^−1^ α-toxin. Therefore, in this study, following experiments were performed by using 100 ng ml^−1^ of α-toxin. Immunoblotting analysis revealed that expression of G-CSFR was decreased by α-toxin (Fig. 4c, d). Plasma membrane localization of G-CSFR was observed in control cells, whereas internalization of G-CSFR was detected in α-toxin-treated cells in the presence and absence of G-CSF (Fig. 4e). Furthermore, our results showed that the treatment of Ly-6G^+^ cells with G-CSF alone decreased the expression of G-CSFR (Fig. 4c–e). Together, the results demonstrated that α-toxin makes neutrophils insensitive to G-CSF by inducing the degradation of its receptor, which would be involved in impaired granulopoiesis by α-toxin.

Next, we tested whether production of ceramide by α-toxin induces the degradation of G-CSFR. Immunofluorescent analysis revealed that α-toxin stimulated the formation of ceramide in Ly-6G^+^ neutrophils (Fig. 4f). C_2_-ceramide dose-dependently inhibited the accelerated cell proliferation by G-CSF and decreased expression of G-CSFR (Fig. 4g–i). Thus stimulation of ceramide production by α-toxin should be involved in the degradation of G-CSFR in α-toxin-treated neutrophils.

### α-Toxin amplifies LPS-induced inflammatory responses

**Fig. 5:**
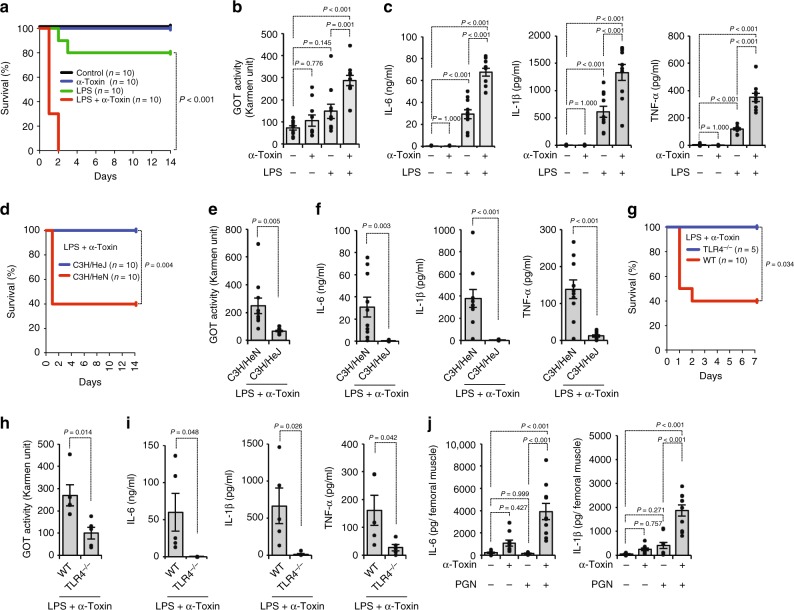
α-Toxin augments Toll-like receptor (TLR)-mediated inflammatory responses. **a**–**c** C57BL/6J mice were injected intraperitoneally with 40 ng of purified α-toxin and 100 μg of lipopolysaccharide (LPS). The survival of mice was monitored, and Kaplan–Meier survival curves are shown (**a**). At 12 h after administration, plasma glutamic-oxaloacetic transaminase (GOT) activities (**b**, *n* = 10 per condition), interleukin (IL)-6 levels, IL-1β levels, and tumor necrosis factor (TNF)-α levels (**c**, *n* = 10 per condition) were determined. **d**–**f** C3H/HeJ mice and C3H/HeN mice were injected intraperitoneally with 40 ng of purified α-toxin and 100 μg of LPS. The survival of mice was monitored, and Kaplan–Meier survival curves are shown (**d**). At 12 h after the administration, plasma GOT activities (**e**, *n* = 10 per condition), IL-6 levels, IL-1β levels, and TNF-α levels (**f**, *n* = 10 per condition) were determined. **g**–**i** C57BL/6J mice (WT) and *Tlr4*^−*/*−^ mice (TLR4^−/−^) were injected intraperitoneally with 40 ng of purified α-toxin and 100 μg of LPS. The survival of mice was monitored, and Kaplan–Meier survival curves are shown (**g**). At 12 h after the administration, plasma GOT activities (**h**, *n* = 5 per condition), IL-6 levels, IL-1β levels, and TNF-α levels (**i**, *n* = 5 per condition) were determined. **j** Mice were injected intramuscularly with 20 ng of purified α-toxin and 100 μg of peptidoglycan (PGN). At 24 h after the administration, IL-6 and IL-1β levels in the muscle were determined by enzyme-linked immunosorbent assay (*n* = 10 per condition). Log-lank test (**a**, **d**, **g**), one-way analysis of variance (**b**, **c**, **j**), and two-tailed Student’s *t* test (**e**, **f**, **h**, **i**) were employed to assess statistical significance. Values are mean ± standard error. Combined data from two independent experiments were shown

The findings about the promotion of G-CSF production by α-toxin prompted us to hypothesize that α-toxin modulates TLR-mediated inflammatory responses. As shown in Fig. 5a, administration of a sub-lethal dose of α-toxin greatly increased the lethal toxicity of LPS. Additionally, plasma levels of a tissue injury marker, glutamic-oxaloacetic transaminase (GOT) activity, were elevated in mice simultaneously administrated with α-toxin and LPS (Fig. 5b). Of note, administration of α-toxin alone resulted in no effect on plasma concentrations of inflammatory cytokines, such as IL-6, IL-1β, and TNF-α, whereas α-toxin greatly promoted LPS-mediated production of these cytokines (Fig. 5c). C3H/HeJ mice carry a missense mutation in the third exon of the TLR4 gene, resulting in their hyporesponsiveness to LPS^43^. The lethal toxicity of simultaneous administration of α-toxin and LPS was not observed in C3H/HeJ mice, whereas six out of ten control mice (C3H/HeN mice) died after the administration (Fig. 5d). LPS and α-toxin-mediated increases in plasma GOT activity and plasma concentration of IL-6, IL-1β, and TNF-α were extraordinarily decreased in C3H/HeJ mice compared with C3H/HeN mice (Fig. 5e, f). These results demonstrated that α-toxin amplifies LPS-induced inflammatory responses in a TLR4-dependent manner. It was further determined that toxicity of simultaneous administration of α-toxin and LPS was not observed in *Tlr4*^−*/*−^ mice, which supports this notion (Fig. 5g–i). Finally, we tested whether α-toxin amplifies PGN-induced inflammatory responses in the skeletal muscle. An intramuscular injection of purified α-toxin alone had no profound impact on the peripheral levels of IL-6 and IL-1β, but simultaneous administration of both agents notably increased them (Fig. 5j). TNF-α was not detectable in any condition (Supplementary Data 1). Thus α-toxin augmented TLR-mediated inflammatory response.

## Discussion

During bacterial infection, G-CSF produced by endothelial cells accelerates granulopoiesis to strengthen host defense, but *C. perfringens* α-toxin impairs granulopoiesis via an unknown mechanism. Here we tested whether α-toxin impedes G-CSF-mediated granulopoiesis and found that α-toxin had no inhibitory effect on the production of G-CSF, but the toxin inhibited G-CSF-mediated cell proliferation of Ly-6G^+^ neutrophils by inducing the degradation of G-CSFR. It has been reported that binding of G-CSF to its receptor induces the internalization of G-CSFR and its degradation to prevent the sustained activation of signaling^44^. Correspondingly, our results showed that the treatment of Ly-6G^+^ cells with G-CSF decreased the expression of G-CSFR. It was interesting that α-toxin induced the internalization of G-CSFR without its activation. Bacterial SMase can hydrolyze cellular membrane sphingomyelin to ceramide^45^, and ceramide has been identified as a modulator of endocytosis^46^. Once a cell membrane is injured, extracellularly released acid SMase converts plasma membrane sphingomyelin to ceramide, and the produced ceramide self-associates to bud into the intracellular space leading to the generation of endosomes, which contributes to repair of the injured plasma membrane^47^. Thus ceramide can modulate endocytosis, so the production of ceramide by the SMase activity of α-toxin on its own might induce the internalization of G-CSFR.

Activation of TLR should be tightly regulated to avoid tissue damage by inappropriate inflammation^48^. Many negative regulators of TLR signaling, including ubiquitin ligases, splice variants for adaptors, and transcriptional regulators, have been identified^48^. Recently, lipid-modifying GPI-anchored sphingomyelin phosphodiesterase, acid-like 3B (SMPDL3B), which influences the composition of cellular membranes, was identified as a negative regulator of TLR signaling^49^. Deficiency of SMPDL3B in macrophages enhanced the inflammatory response upon stimulation of TLR. In addition, *Smpdl3b*^−*/*−^ mice displayed augmented responsiveness to TLR stimulation in peritonitis models. Components of cellular membranes, such as ceramide and cholesterol, have been implicated in the regulation of TLR signaling^50^. Macrophages deficient in cholesterol efflux transporter ATP-binding cassette transporter A1 (ABCA1) have enlarged lipid rafts and exhibit hyper-responsive to LPS^51^. Thus modification of the cellular membrane affects the activity of TLR signaling, which is a crucial regulatory mechanism of inflammatory responses during bacterial infection. As described above, bacterial SMase hydrolyzes sphingomyelin to ceramide on the cell membrane^45^. Recently, we reported that α-toxin disturbs lipid raft integrity in an enzyme activity-dependent manner^33^. Furthermore, α-toxin induces the clustering of GM1 ganglioside, which is a component of lipid rafts^52^, on the cell membrane in a lung adenocarcinoma epithelial cell line, A549 cells^53^. The findings suggested that the SMase activity of α-toxin induces an alteration in the membrane lipid composition and modification of membrane lipid fluidity, which would be involved in the amplification of TLR signaling by the toxin. However, C_2_-ceramide had no impact on the production of G-CSF in PGN-stimulated HUVECs, so it should be borne in mind that the amount of ceramide does not simply determine how much inflammatory response occurs. We also found that activation of endogenous PLCβ-3 by α-toxin contributed to increased production of G-CSF from endothelial cells. Further studies are needed to unveil how α-toxin modifies TLR signaling.

**Fig. 6:**
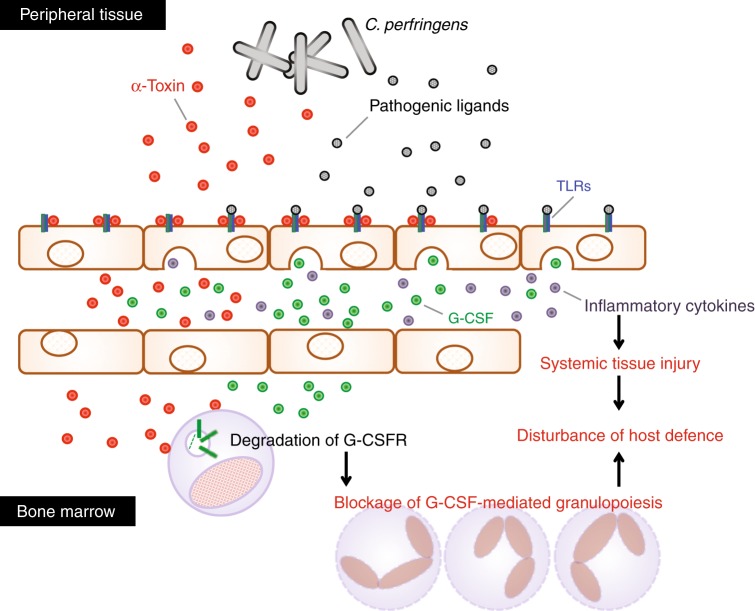
Model of disturbed host defense by blockage of granulocyte colony-stimulating factor (G-CSF)-mediated granulopoiesis and Toll-like receptor (TLR)-mediated overproduction of inflammatory cytokines in a *C. perfringens*-infected host. α-Toxin upregulates the production of G-CSF from endothelial cells by promoting pathogenic ligand-induced TLR signaling, but the toxin makes neutrophils insensitive to G-CSF by inducing the degradation of its receptor, which could be relevant to the α-toxin-mediated blockage of granulopoiesis. Furthermore, α-toxin augmented the TLR-mediated inflammatory response, resulting in systemic and/or local tissue injury through the overproduction of inflammatory cytokines. Thus α-toxin disturbs host defense by modulating G-CSF receptor (G-CSFR)-mediated granulopoiesis and TLR-mediated inflammation

The host innate immune system is precisely regulated through G-CSF-mediated granulopoiesis^4^; however, some bacteria overwhelm the immune system to cause serious and life-threatening infection. We reported that *C. perfringens* α-toxin interfered with the replenishment of mature neutrophils in the peripheral circulation leading to impairment of the innate immune system^31^. Additionally, an absence of polymorphonuclear leukocytes at the site of *C. perfringens* infection has been reported^20,21^. Moreover, α-toxin promotes the formation of platelet–leukocyte aggregates accompanied by vascular occlusion and a marked reduction in microvascular perfusion, resulting in impeded neutrophil extravasation into peripheral tissue^23–25^. Thus *C. perfringens* α-toxin impairs neutrophil-mediated innate immune system. In the present study, we tested whether α-toxin disturbs G-CSF-mediated granulopoiesis and found that α-toxin upregulates the production of G-CSF from endothelial cells by promoting pathogenic ligand-induced TLR signaling in vivo and in vitro, whereas α-toxin makes neutrophils insensitive to G-CSF by reducing the expression of its receptor, which could be relevant to the α-toxin-mediated blockage of granulopoiesis (Fig. 6). It is possible that the augmented production of G-CSF is a consequence of the host response to strengthen granulopoiesis. Unknown mechanisms sensing pathogenic bacterial toxins to modulate TLR signaling might exist. In addition, administration of α-toxin promoted LPS-induced lethality and tissue injury accompanied by accelerated production of inflammatory cytokines (Fig. 6). Similarly, α-toxin amplified PGN-induced production of inflammatory cytokines in the skeletal muscle. Clinically, it is a pertinent idea that patients are exposed to a broad range of bacteria with no discrimination between Gram-negative and Gram-positive bacteria. Indeed, a broad range of pathogenic bacteria has been identified in *C. perfringens*-infected patients, which means that polymicrobial infection occurs in clinical situations^54^. Additionally, high pathogenicity and mortality has been reported in severe diarrhea in piglets caused by co-infection of *C. perfringens* type A and *Escherichia coli*^55^. Thus the activation of not only TLR2 but also TLR4 by α-toxin should be important to understand clinical features of *C. perfringens* infection. Moreover, α-toxin can be detected in bone marrow in mice intramuscularly injected with *C. perfringens*^31^, which means α-toxin spreads to distant organs. The finding suggests that α-toxin has possibility to come in contact with LPS-stimulated cells, such as intestinal epithelial cells. Together, it should be meaningful to speculate the role of α-toxin in TLR4-mediated inflammatory response in *C. perfringens* infection. Yang et al. reported that most patients with *C. perfringens* bacteremia presented initially with systemic inflammation such as fever, leukocytosis, anemia, and thrombocytopenia^56^. The augmented cytokines release by α-toxin might contribute to the characteristics of *C. perfringens* infection, such as the destruction of muscle, shock, multiple organ failure, systemic inflammation, and death of patients^19^. In conclusion, our results illustrated that α-toxin disturbs host defense by modulating G-CSFR-mediated granulopoiesis and TLR-mediated inflammation, which offers a perspective to elucidate how pathogenic bacteria disrupt and evade the host immune system.

## Methods

### Mice

C57BL/6J, C3H/HeJ, and C3H/HeN mice were purchased from SLC (Shizuoka, Japan). For all experiments, female mice aged >7 weeks were used. These mice were kept in a specific pathogen-free animal facility at Tokushima Bunri University. Experiments were approved by the Animal Care and Use Committee of Tokushima Bunri University. The procedures were performed in accordance with institutional guidelines that conform to the Fundamental Guidelines for Proper Conduct of Animal Experiment and Related Activities in Academic Research Institutions under the jurisdiction of the Ministry of Education, Culture, Sports, Science and Technology, 2006.

Femurs and tibias derived from *Tlr2*^−*/*−^ (C57BL/6)^13^, *Tlr4*^−*/*−^ (C57BL/6)^57^, and *Myd88*^−*/*−^ (C57BL/6)^58^ were purchased from Oriental Bio Service, Inc. (Kyoto, Japan). In vivo experiments using *Tlr4*^−*/*−^ mice were carried out in Oriental Bio Service, Inc.

### Reagents and strains

Fluorescein isothiocyanate (FITC)- or phycoerythrin (PE)-conjugated specific antibodies against mouse CD11b (clone M1/70) (1:20 dilution) or Ly-6G (clone 1A8) (1:20 dilution), purified rat anti-mouse CD16/CD32 (Fc Block) (1:100 dilution), and an antibody against CD31 (1:100 dilution) were purchased from BD Biosciences (CA, USA). Specific antibodies against MEK1/2 (1:1000 dilution), phospho-MEK1/2 (1:1000 dilution), JNK (1:1000 dilution), phospho-JNK (1:1000 dilution), p-38 MAPK (1:1000 dilution), phospho-p-38 MAPK (1:1000 dilution), ERK1/2 (1:1000 dilution), phospho-ERK1/2 (1:1000 dilution), PLCγ-1 (1:1000 dilution), phospho-PLCγ-1 (1:1000 dilution), PLCγ-2 (1:1000 dilution), phospho-PLCγ-2 (1:1000 dilution) or IκBα (1:1000 dilution), horseradish peroxidase-linked antibodies against mouse IgG (1:2000 dilution) and rabbit IgG (1:2000 dilution), and U0126 were purchased from Cell Signaling Technology (MA, USA). An antibody against phospho-PLCβ-3 (1:1000 dilution) and β-actin (1:2000 dilution) were from Santa Cruz Biotechnology (CA, USA). Specific antibodies against G-CSFR (1:1000 dilution) and G-CSF (1:100 dilution), Alexa Fluor 488 goat anti-rat IgG (1:1000 dilution), Alexa Fluor 488 goat anti-mouse IgG (1:1000 dilution), U73122, and U73343 were obtained from Abcam (MA, USA). Mouse G-CSF was from Miltenyi Biotec (Bergisch Gladbach, Germany). GDC-0994, FR180204, JNK-IN-8, and SP600125 were purchased from MedChem Express (NJ, USA). PGN from *Bacillus subtilis*, LPS from *E. coli* O111:B4, and specific antibodies against α-tubulin and ceramide, C_2_-ceramide, and FITC-conjugated dextran (average molecular weight = 10 kDa) were from Sigma-Aldrich (MO, USA). Pam_3_CSK_4_ and fibroblast-stimulating lipopeptide were obtained from Abcam (MA, USA). To assess plasma GOT activity or creatine kinase activity, commercial assay kits, Transaminase CII test Wako (Wako Pure Chemical Industries, Osaka, Japan) or Creatine Kinase Activity Assay Kit (Abcam, MA, USA), was used, respectively. All other chemicals were of the highest grade available from commercial sources. *C. perfringens* WT Strain 13 and *B. subtilis* ISW1214 were used in this study. Preparation of a *plc* gene-knockout mutant of *C. perfringens* (PLC-KO) was described in our previous report^31^.

### Bacterial culture and infection

Bacterial culture and infection were performed as previously described^31^. Briefly, *C. perfringens* WT strain 13 or PLC-KO were grown in TGY (tryptone, glucose, and yeast extract) medium in anaerobic conditions at 37 °C. Exponentially growing bacteria were harvested, washed, re-suspended in TGY medium, and injected into the femoral muscle of mice. To quantify CFUs, residual bacteria were serially diluted, plated on brain heart infusion agar plates, and cultured anaerobically at 37 °C.

### Purification of WT and variant α-toxin

As described previously, purification of WT or H148G variant α-toxin was performed^59,60^. Briefly, recombinant forms of pHY300PLK harboring the structural genes of WT or H148G variant α-toxin were introduced into *B. subtilis* ISW1214 by transformation, and the transformants were cultured in Luria-Bertani broth containing 50 μg ml^−1^ ampicillin at 37 °C. Next, the culture medium was collected, and α-toxin secreted into the culture medium was purified chromatographically.

### BMC isolation and culture

Isolation of BMCs was as described in a previous report^31^. To isolate BMCs, femurs and tibias were crushed in phosphate-buffered saline (PBS) supplemented with 2% heat-inactivated fetal bovine serum (FBS; AusGeneX, QLD, Australia), and filtered through a 40-μm mesh. Red blood cells were hemolyzed with lysis buffer (ACK lysing buffer; GIBCO, NY, USA). The number of living cells was counted after trypan blue staining. Isolated cells were cultured in RPMI 1640 medium supplemented with 10% FBS, 100 units ml^−1^ penicillin, and 100 μg ml^−1^ streptomycin at 37 °C.

### Flow cytometric analysis

Flow cytometric analysis was performed as described previously^31^. After blocking Fc-receptors with purified rat anti-mouse CD16/CD32, cells were labeled with antibodies diluted in PBS containing 2% FBS. The labeled cells were analyzed using a Guava easyCyte (Millipore, MA, USA). Data were analyzed using the FlowJo software (Tree Star, OR, USA).

### Magnetic cell isolation

As described previously^16^, Ly-6G^+^ cells were isolated using an EasySep system (StemCell Technologies, BC, Canada) in accordance with the manufacturer’s protocol. Briefly, Ly-6G^+^ cells were labeled with the PE-conjugated specific antibodies followed by antibody conjugation to magnetic nanoparticles using EasySep PE Selection cocktail. The labeled cells were separated using EasySep Magnet (StemCell Technologies, BC, Canada).

Isolated Ly-6G^+^ cells were cultured in RPMI 1640 medium supplemented with 1% or 10% FBS, 100 units ml^−1^ penicillin, and 100 μg ml^−1^ streptomycin. The amount of viable cells was determined using a Cell Counting Kit-8 cell viability assay in accordance with the manufacturer’s instructions (Dojindo, Kumamoto, Japan).

### Enzyme-linked immunosorbent assay (ELISA)

ELISAs were performed as described previously^16^. Using heparinized syringes, peripheral blood was obtained via the vena cava from mice. To dissociate femoral muscle, isolated muscle was cut into small pieces in PBS and dissociated in a gentleMACS C tube using a gentle MACS dissociator (Miltenyi Biotec, Bergisch Gladbach, Germany). Measurement of G-CSF, IL-6, IL-1β, and TNF-α levels was performed using mouse Quantikine ELISA kits in accordance with the manufacturer’s instructions (R&D Systems, MN, USA).

HUVECs were purchased from PromoCell, and the cells were cultured in Endothelial Cell Growth Medium 2 (PromoCell) in accordance with the manufacturer’s protocols. After treatment of the cells with α-toxin and PGN, the culture supernatants were harvested, and the G-CSF levels were measured using a human G-CSF Quantikine ELISA Kit (R&D Systems, MN, USA).

### Immunohistochemistry and immunofluorescence microscopy

Ly-6G^+^ cells treated with α-toxin and G-CSF were cytospinned onto microscopic glass slides and blocked with Blocking One Histo (Nacalai Tesque, Inc., Kyoto, Japan). The samples were then incubated with a primary antibody against G-CSFR. After washing with PBS, samples were incubated with the secondary antibody conjugated with Alexa Fluor 488.

Femoral muscles were embedded in OCT compound (Sakura Finetek Japan, Tokyo, Japan), and cryosectioning of the frozen tissue was performed using a cryostat microtome (Leica, IL, USA). Sections were blocked with Blocking One Histo and incubated with primary antibodies. Finally, the sections were incubated for 1 h with Alexa Fluor 546 goat anti-rabbit IgG and Alexa Fluor 488 goat anti-rat IgG. The antibodies were diluted in DAKO Antibody Diluent (DAKO, Glostrup, Denmark). Nuclei were stained with 4’,6-diamino-2-phenylindole. Images were captured on a confocal laser-scanning fluorescence microscope (Nikon A1, Nikon instruments, Tokyo, Japan).

### Immunoblotting analysis

Immunoblotting analysis was performed as described previously with some modifications^61^. Ly-6G^+^ cells or HUVECs were lysed in RIPA buffer (Nacalai Tesque, Inc., Kyoto, Japan) or Nonidet P-40 (NP-40) lysis buffer (50 mM Tris-HCl, pH 8.0, 150 mM NaCl, 1% NP-40), respectively. These buffers were supplemented with protease inhibitor cocktail and phosphatase inhibitor cocktail (Nacalai Tesque, Inc., Kyoto, Japan). The protein concentrations of the samples were determined using a Protein Assay Bicinchoninate Kit (Nacalai Tesque, Inc., Kyoto, Japan). Samples were applied to 10% polyacrylamide gels containing sodium dodecyl sulfate, subjected to electrophoresis, and transferred to a polyvinylidene difluoride membrane (Immobilon P; Millipore, MA, USA). The membrane was blocked with Blocking One or Blocking One-P (Nacalai Tesque, Inc., Kyoto, Japan). The proteins were immunoblotted with each antibody.

### Real-time reverse transcriptase PCR (RT-PCR) analysis

Total RNA was extracted using an RNeasy Mini Kit (QIAGEN, Hilden, Germany) in accordance with the manufacturer’s protocol, and the samples were reverse-transcribed using Superscript III First-Strand Synthesis SuperMix (Invitrogen, MD, USA). Synthesized cDNA was used in real-time RT-PCR (StepOnePlus Realtime-PCR System; Applied Biosystems, CA, USA) experiments using gene-specific primers and iQ SYBR GREEN Supermix (Bio-Rad, CA, USA). Relative mRNA expression was calculated using standard curves. Results were normalized to the level of β-actin. Primer sequences are shown in Table 1.

**Table 1:** List of primer sequences used for real-time RT-PCR analysis

Gene	Forward (5’ → 3’)	Reverse (5’ → 3’)
*ACTB* (β-actin)	AGAGCTACGAGCTGCCTGAC	AGCACTGTGTTGGCGTACAG
*CSF3* (G-CSF)	GCTGTGCCACCCCGAGG	CAGGAGCCCCTGGTAGAG

### Dextran exocytosis

Exocytosis of dextran was assessed as described previously with some modifications^62^. HUVECs were incubated for 4 h with FITC-conjugated dextran (0.5 mg ml^−1^) to complete endocytosis. The cells were washed three times with Endothelial Cell Growth Medium 2, and the incubation of the cells was started. After 4 or 24 h, the fluorescence intensity of FITC-dextran in the culture supernatants was measured (excitation, 488 nm; emission, 530 nm), and the concentration of FITC-dextran was calculated using a standard curve.

### Dissociation of *C. perfringens*-infected femoral muscle

*C. perfringens*-infected femoral muscle was isolated 24 h after the infection. Isolated muscle was cut into small pieces of 2–4 mm in TGY medium and dissociated in a gentleMACS C tube (Miltenyi Biotec) using a gentle MACS dissociator (Miltenyi Biotec) as described previously^31^. The supernatant was serially diluted, plated on BHI agar plates, and cultured anaerobically at 37 °C.

### Isolation of bone marrow-derived monocytes

For the isolation of Ly-6G^−^Ly-6C^+^ monocytes, we performed two-step separation as described previously^16^. Briefly, Ly-6G^+^ cells were labeled with the PE-conjugated specific antibody against Ly-6G, and the negative fraction was magnetically purified using EasySep PE Selection cocktail. Next, the cells were labeled with an FITC-conjugated specific antibody against Ly-6C followed by antibody conjugation to magnetic nanoparticles using EasySep FITC Selection cocktail. The positive fraction was obtained as Ly-6G^−^Ly-6C^+^ cells.

### Statistical analysis

All statistical analyses were performed with Easy R (Saitama Medical Center, Jichi Medical University, Saitama, Japan)^63^. Differences between two groups were evaluated using two-tailed Student’s *t* test. One-way analysis of variance followed by Tukey’s test was used to evaluate differences among three or more groups. Differences in survival rate of mice were determined by Kaplan–Meier analysis and evaluated by log-lank test. Differences were considered to be significant for values of *P* < 0.05.

## Supplementary information

Supplementary Information

Description of Additional Supplementary Files

Supplementary Data 1

## Data Availability

Raw gel images are available in Supplementary Figure 9. The data source underlying the graphs in the main figures is available in Supplementary Data 1.
