# Supplementary material for: Clostridium perfringens α-toxin impairs granulocyte colony-stimulating factor receptor-mediated granulocyte production while triggering septic shock
Source: Commun Biol. 2019 Jan 31;2:45. doi: 10.1038/s42003-019-0280-2 (PMC6355902; doi:10.1038/s42003-019-0280-2)
Supplement: Supplementary file 1 — Supplementary Information [file 42003_2019_280_MOESM1_ESM.pdf]

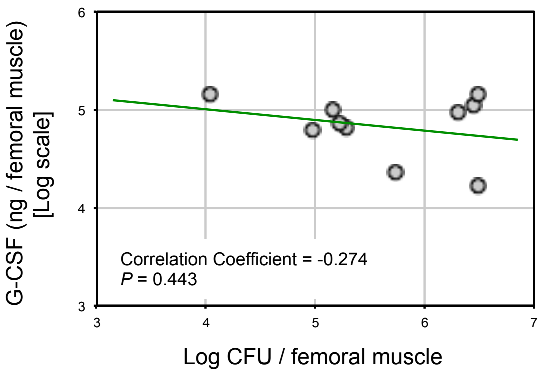

Supplementary Figure 1. Relationship between G-CSF production and bacterial CFUs in *C. perfringens*-infected muscle. Mice were intramuscularly injected with 1 × 10^7 CFUs of *C. perfringens* Strain 13. At 24 hours after the infection, G-CSF levels in the infected muscle were measured by ELISA, and *C. perfringens* CFUs in the muscle were determined as described in Supplementary Methods. Pearson correlation coefficient between the G-CSF levels and bacterial CFUs was determined.

a

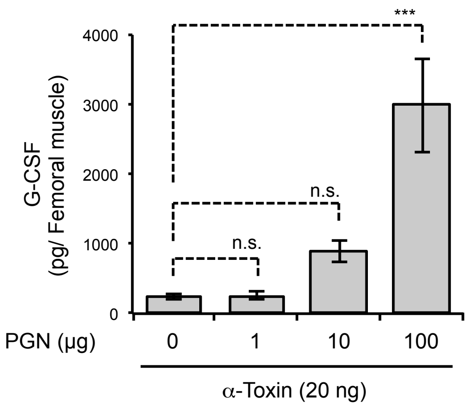

b

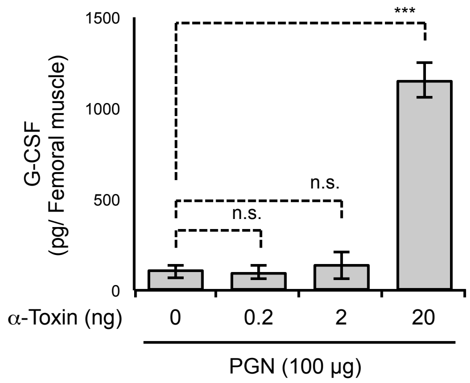

Supplementary Figure 2. A dose response of α-toxin and PGN for G-CSF production in mouse muscle. Mice were injected intramuscularly with the indicated amount of α-toxin and PGN. At 24 hours after the administration, G-CSF levels in the muscle were determined.

One-way ANOVA was employed to assess statistical significance. Values are mean ± standard error. ***$P < 0.001$; n.s., not significant.

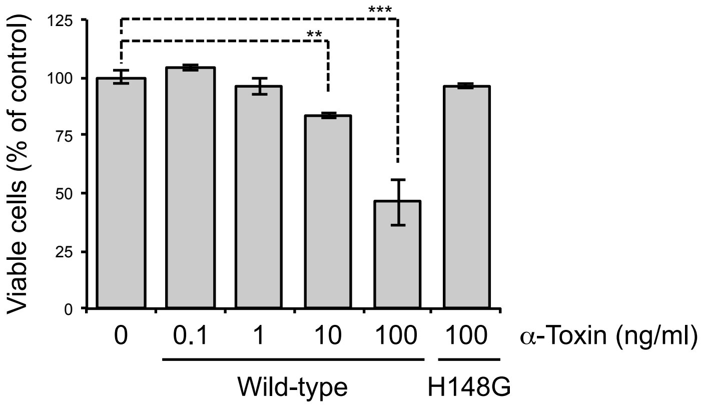

Supplementary Figure 3. *C. perfringens* α-toxin-induced cytotoxicity for HUVECs. HUVECs were cultured for 24 hours in the presence or absence of the indicated concentrations of α-toxin (Wild-type) or a variant α-toxin (H148G). Viable cells were determined using a Cell-Counting Kit-8.

One-way ANOVA was employed to assess statistical significance. Values are mean ± standard deviation. $^{**}P < 0.01$; $^{***}P < 0.001$.

a

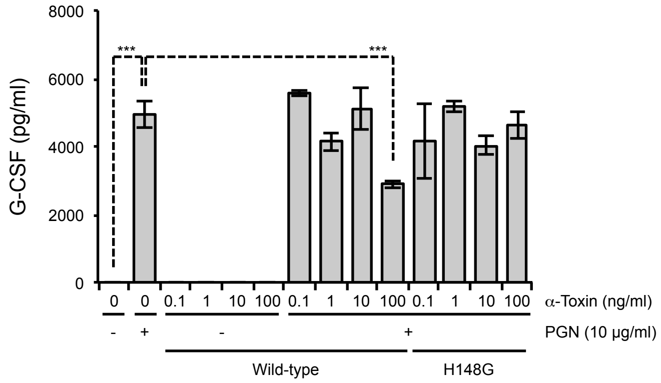

b

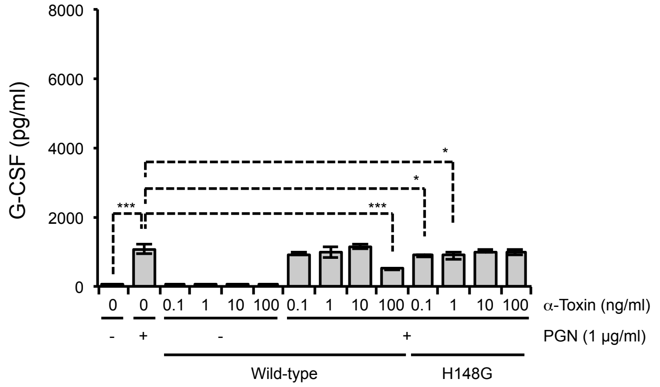

Supplementary Figure 4. *C. perfringens* α-toxin does not accelerate the production of G-CSF in bone marrow-derived monocytes. Magnetically isolated Ly-6G$^-$Ly6C$^+$ monocytes were cultured for 24 hours in the presence or absence of the indicated concentrations of α-toxin (Wild-type) or a variant α-toxin (H148G), and PGN. G-CSF levels in the culture medium were determined by ELISA.

One-way ANOVA was employed to assess statistical significance. Values are mean ± standard deviation. **P* < 0.05; ****P* < 0.001.

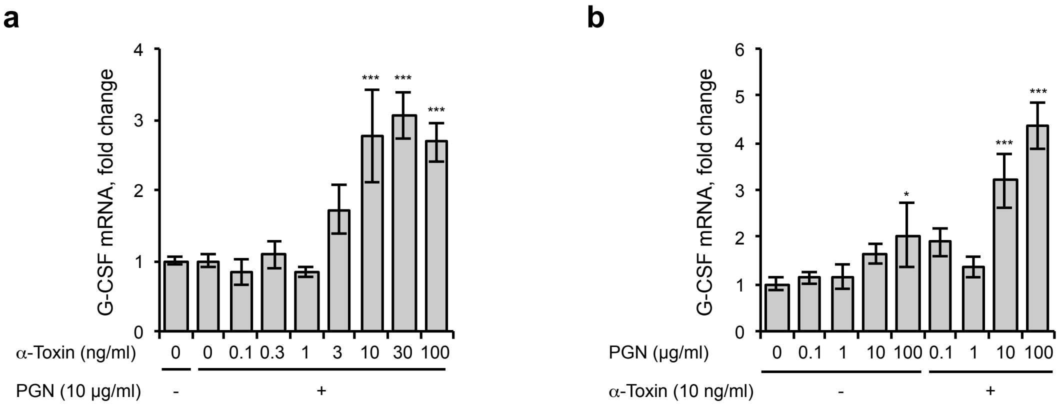

Supplementary Figure 5. A dose response of α-toxin and PGN for G-CSF production in HUVECs. HUVECs were cultured for 4 hours in the presence or absence of the indicated concentration of α-toxin and PGN. Total RNA was extracted and subjected to real-time RT-PCR using a specific primer set for G-CSF.

One-way ANOVA was employed to assess statistical significance. Values are mean ± standard deviation. $*P < 0.05$, compared with control; $***P < 0.001$, compared with control.

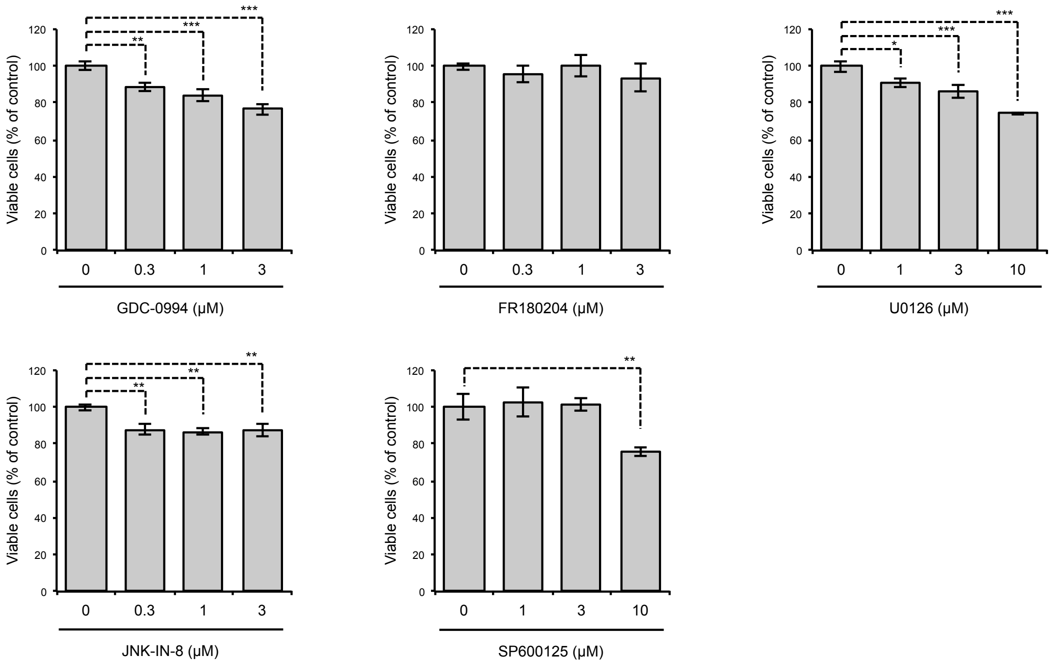

Supplementary Figure 6. Viability of HUVECs treated with various inhibitors. HUVECs were cultured for 24 hours in the presence of 10 ng/ml α-toxin and 10 μg/ml PGN, and in the presence or absence of the indicated concentrations of GDC-0994, FR180204, U0126, JNK-IN-8, or SP600125. Viable cells were determined using a Cell-Counting Kit-8.

One-way ANOVA was employed to assess statistical significance. Values are mean ± standard deviation. $*P < 0.05$; $**P < 0.01$; $***P < 0.001$.

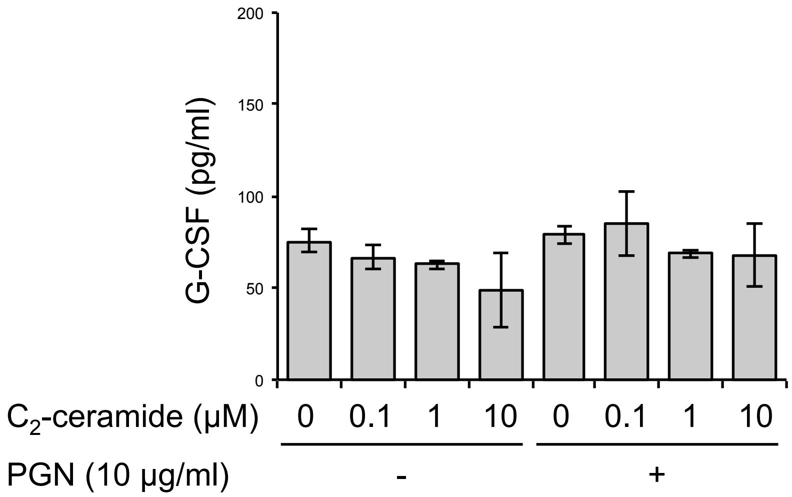

Supplementary Figure 7. C_2-ceramide has no impact on production of G-CSF in HUVECs. HUVECs were cultured for 24 hours in the presence or absence of the indicated concentration of C_2-ceramide and 10 μg/ml PGN. G-CSF levels in the culture medium were determined.

One-way ANOVA was employed to assess statistical significance, and there was no significant difference between control and C_2-ceramide-treated groups. Values are mean ± standard deviation.

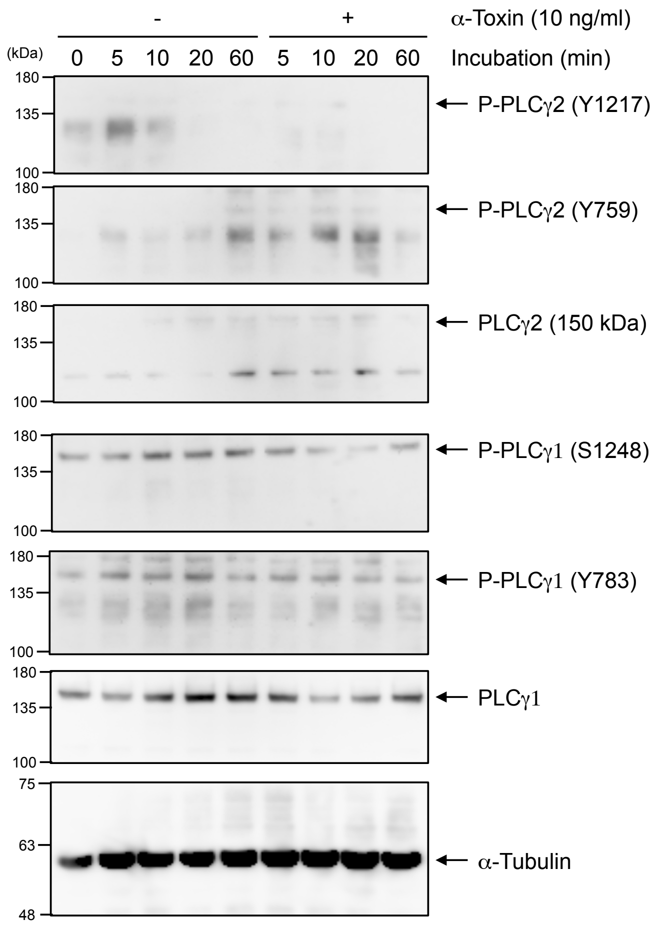

Supplementary Figure 8. α-Toxin has no impact on phosphorylation of PLCγ-1 and PLCγ-2. HUVECs were cultured in the presence or absence of α-toxin, and whole cell extracts were analyzed at the indicated time by immunoblotting with specific antibodies.

Figure 2c

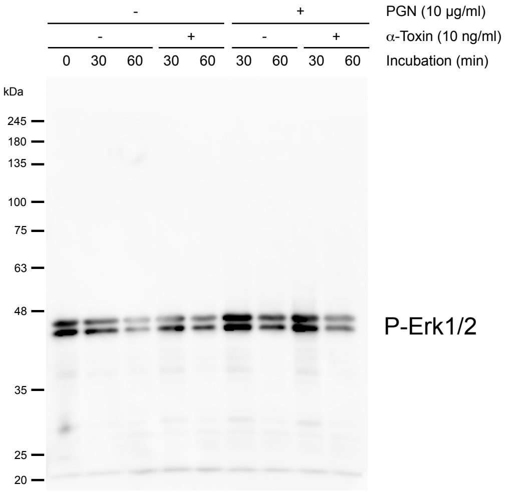

Figure 2c

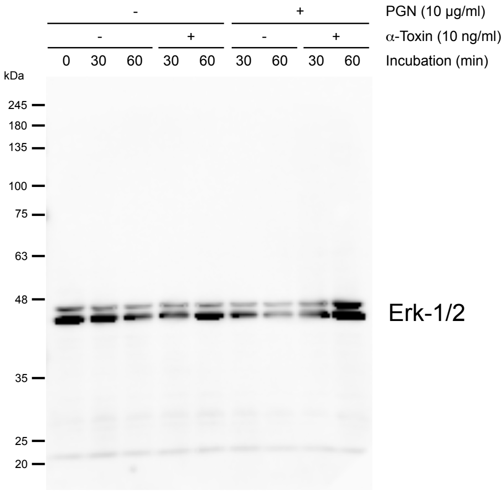

Figure 2c

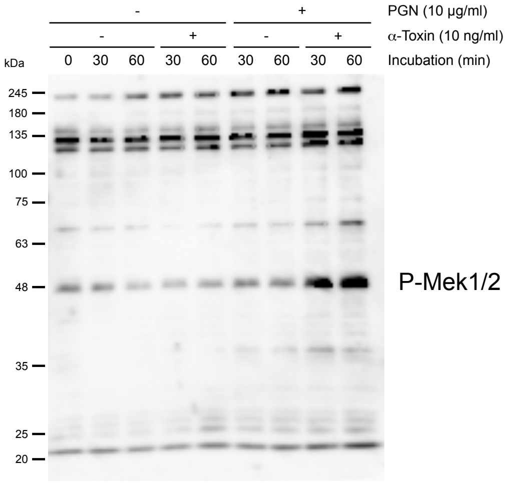

Figure 2c

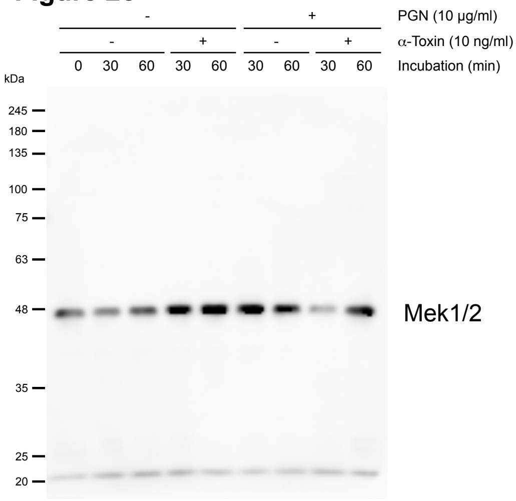

Supplementary Figure 9. Original immunoblots.

Figure 2c

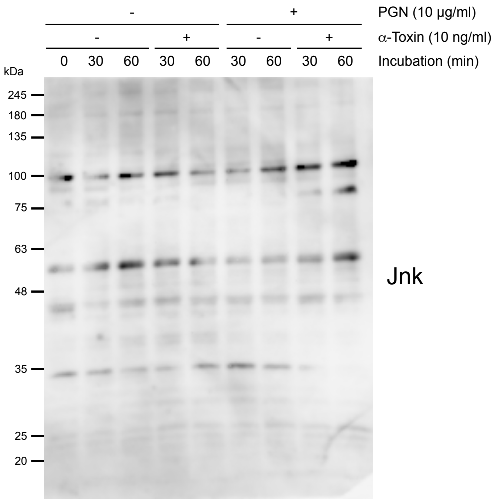

Figure 2c

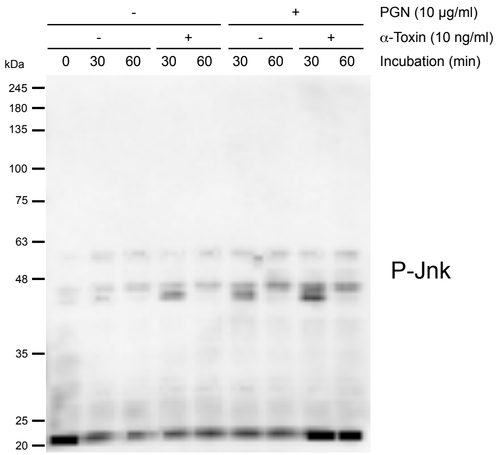

Figure 2c

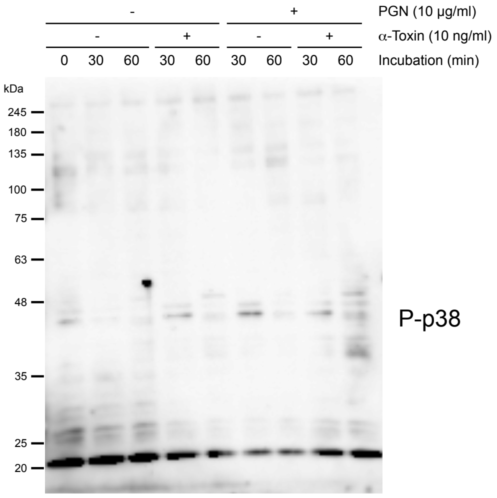

Figure 2c

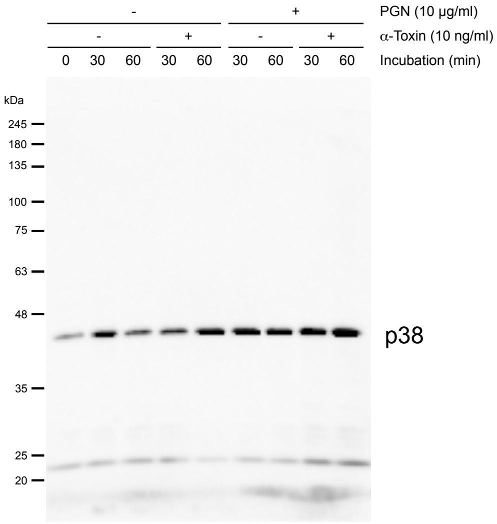

Supplementary Figure 9 (continued). Original immunoblots.

Figure 2c

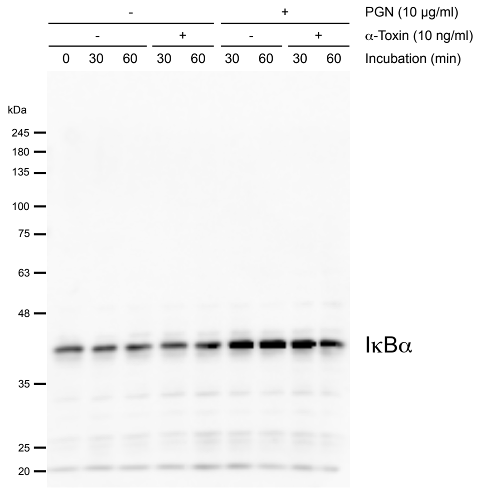

Figure 2c

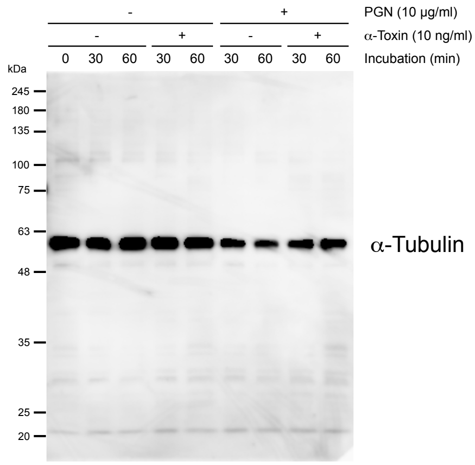

Figure 3a

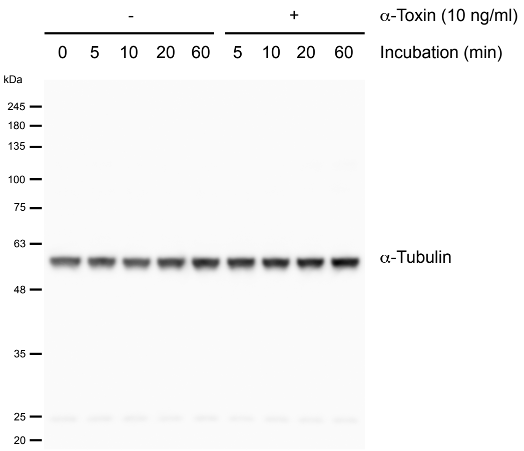

Figure 3a

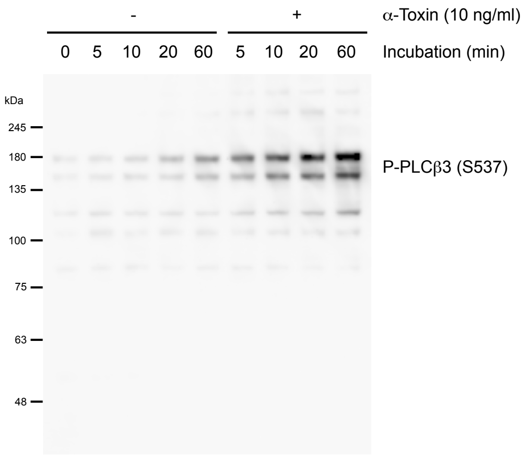

Supplementary Figure 9 (continued). Original immunoblots.

Figure 3c

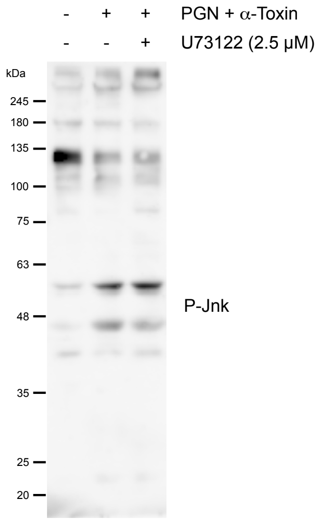

Figure 3c

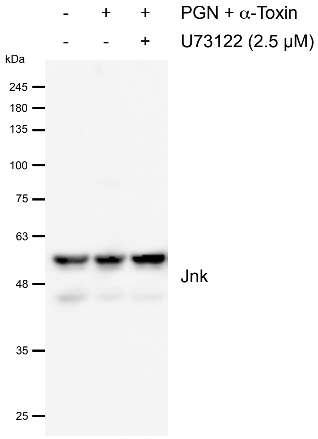

Figure 3c

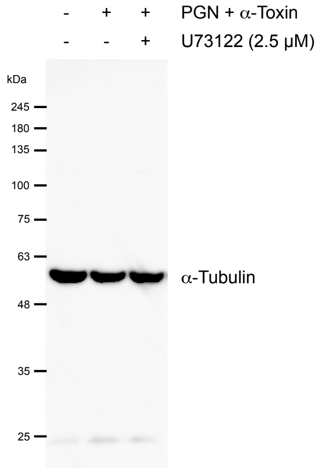

Figure 4c

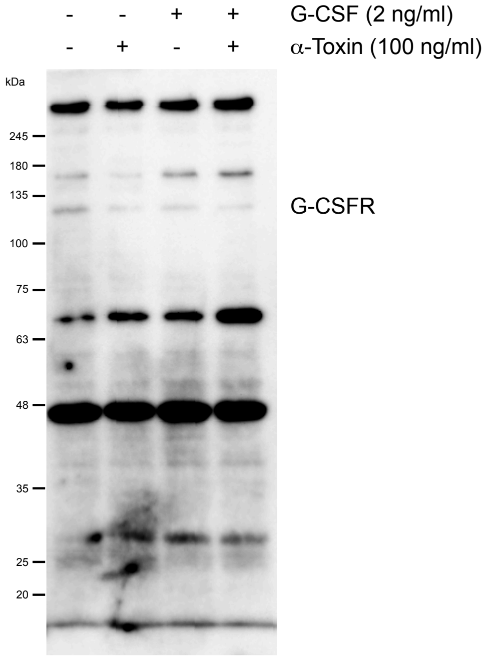

Figure 4c

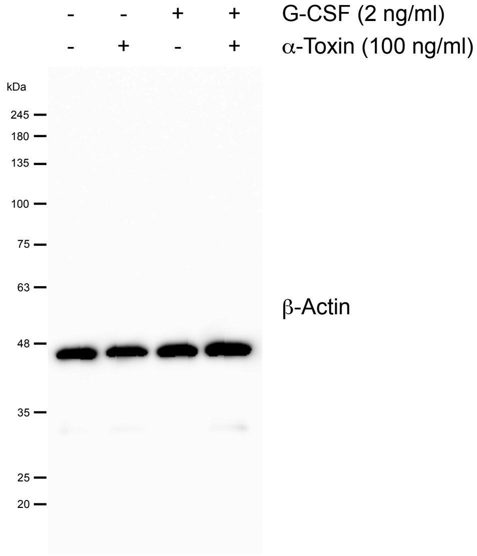

Supplementary Figure 9 (continued). Original immunoblots.

Figure 4h

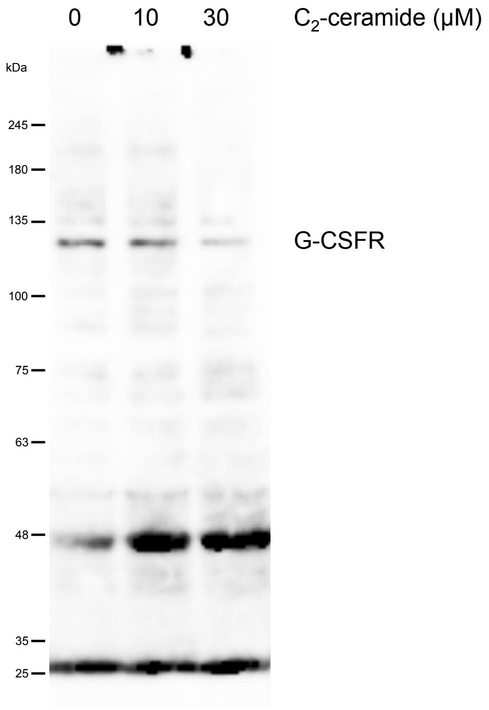

Figure 4h

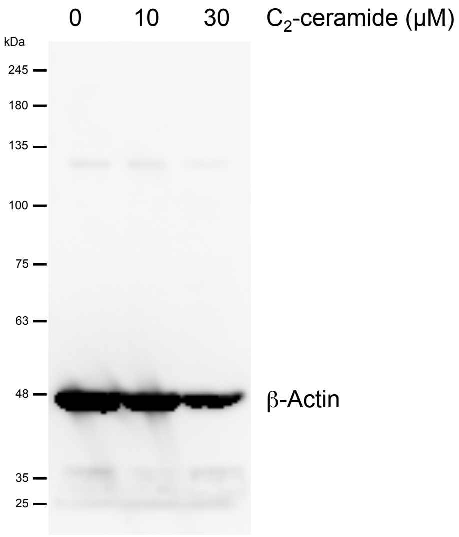

Supplementary Figure 9 (continued). Original immunoblots.
