# Supplementary material for: Clostridium perfringens α-toxin impairs granulocyte colony-stimulating factor receptor-mediated granulocyte production while triggering septic shock
Source: Commun Biol. 2019 Jan 31;2:45. doi: 10.1038/s42003-019-0280-2 (PMC6355902; doi:10.1038/s42003-019-0280-2)
Supplement: Supplementary file 2 — Description of Additional Supplementary Files [file 42003_2019_280_MOESM2_ESM.docx]

**Descriptions of Additional Supplementary File**

**File Name:** Supplementary Data 1

**Description:** The source data underlying the graphs and charts presented in figures. The source data (Protein expressions, Relative protein expressions, Relative gene expressions, Relative amounts of phosphorylated proteins, Proportions of neutrophils in bone marrow cells, Relative cell viabilities, Relative plasma GOT activities) underlying the graphs presented in Figure 1-5 is shown.
